# Incidence, Prognosis, and Management of Occult Endometrial Carcinoma After Hysterectomy for Benign Disease: A Systematic Review and Meta-Analysis

**DOI:** 10.3390/diagnostics16213450

**Published:** 2026-10-22

**Authors:** Ying-Hsi Chen, Dah-Ching Ding

**Affiliations:** 1 Department of Obstetrics and Gynecology, Hualien Tzu Chi Hospital, Buddhist Tzu Chi Medical Foundation, Tzu Chi University, Hualien 970, Taiwan; celestmeroy@gmail.com; 2 Institute of Medical Sciences, Tzu Chi University, Hualien 970, Taiwan

**Keywords:** occult endometrial carcinoma, incidental uterine malignancy, hysterectomy, morcellation, pelvic organ prolapse, meta-analysis

## Abstract

**Background/Objectives**: Occult endometrial carcinoma, incidentally diagnosed after hysterectomy for presumed benign disease, raises concerns regarding its incidence, the prognostic effects of minimally invasive surgery and morcellation, and subsequent management. This systematic review synthesized the available evidence and performed meta-analysis where appropriate. **Methods**: PubMed, Scopus, and the Cochrane Central Register were searched using a sensitivity-tested strategy. After de-duplication and screening, 76 studies met the eligibility criteria. Incidence was pooled using a random-effects meta-analysis of proportions with the Freeman–Tukey double-arcsine transformation. Subgroup analyses examined surgical contexts, including hysterectomy for benign disease, morcellation-specific cohorts, and pelvic organ prolapse surgery. Survival and management outcomes were synthesized narratively. **Results**: Among 68 studies with extractable denominators, the pooled incidence (proportion of hysterectomies with carcinoma on final pathology) of occult endometrial carcinoma following hysterectomy for benign disease was 0.33% (95% prediction interval, 0.04–0.87%). Incidence was lowest in selected morcellation cohorts (0.22%) and higher in unselected benign hysterectomy cohorts (0.33%) and prolapse populations (0.43%; test for subgroup differences, *p* = 0.03), with substantially higher rates among older patients. The pooled incidence of occult uterine malignancies, including sarcoma, was 0.54%. For endometrial carcinoma specifically, the limited observational evidence did not demonstrate a statistically significant association between power morcellation or laparoscopic surgery and poorer survival, although confidence intervals were wide. However, morcellation was associated with increased mortality among patients with uterine sarcoma. Occult endometrial carcinomas were predominantly early-stage and low-grade, with 81.8% classified as International Federation of Gynecology and Obstetrics IA, and approximately 91% achieving 5-year overall survival. In a small series of selected high-risk patients, completion staging resulted in upstaging in 25% and informed adjuvant treatment decisions. Limited observational data did not demonstrate a statistically significant survival benefit of complementary lymphadenectomy, although estimates were imprecise. **Conclusions**: Occult endometrial carcinoma occurs in approximately 0.2–0.4% of hysterectomies performed for benign disease, with a pooled incidence of 0.33% and higher rates among older patients. The limited observational evidence did not demonstrate a statistically significant association between minimally invasive surgery or morcellation and poorer survival, but estimates were imprecise; wide confidence intervals and potential residual confounding preclude conclusions regarding oncologic safety. Overall prognosis was favorable. These findings support age-informed preoperative counseling and appropriate endometrial evaluation, with individualized consideration of re-staging when occult carcinoma is identified.

## 1. Introduction

Hysterectomy is one of the most frequently performed gynecological procedures [1,2]. Most hysterectomies are indicated for leiomyoma, abnormal uterine bleeding, endometriosis, and pelvic organ prolapse (POP) [3,4,5]. On final histopathological examination, a small proportion of specimens are found to contain an unsuspected uterine malignancy. However, “occult uterine malignancy” is an umbrella term for biologically distinct entities: endometrial carcinoma, which arises from the endometrial epithelium, and uterine sarcomas, most notably leiomyosarcoma, which arise from the myometrium or endometrial stroma. Endometrial carcinoma, the most common gynecological cancer in high-income countries [6], is the most frequent histological type among occult uterine malignancies.

Clinical attention to occult malignancy intensified after the 2014 United States Food and Drug Administration safety communication on power morcellation, which highlighted the risk of disseminating occult malignancy during minimally invasive tissue extraction [7,8]. That warning, and much of the subsequent morcellation literature, was driven predominantly by concern about occult uterine sarcoma, particularly leiomyosarcoma, which cannot be reliably distinguished from leiomyoma before surgery and in which morcellation has been associated with higher recurrence and mortality [9,10,11]. By comparison, occult endometrial carcinoma has received less focused attention and is often pooled with sarcoma under the broader heading of occult uterine malignancy. We focused specifically on endometrial carcinoma for three reasons. First, as the most frequent occult uterine malignancy, it accounts for most women who receive an unexpected cancer diagnosis after hysterectomy for benign disease [12]. Second, its biology differs from that of sarcoma: endometrial carcinoma is frequently diagnosed at an early stage with low-grade endometrioid histology [13], so the prognostic consequences of morcellation cannot be extrapolated from sarcoma data, and pooling the two entities may obscure entity-specific effects. Third, unlike sarcoma, endometrial carcinoma can often be detected preoperatively by endometrial sampling and has established surgical staging and adjuvant treatment pathways [14]; an incidental diagnosis, therefore, raises distinct questions about preoperative evaluation, re-staging, and adjuvant therapy [15]. To provide context, occult uterine malignancy overall, including sarcoma, was analyzed as a secondary outcome.

Accordingly, the incidental diagnosis of endometrial carcinoma raises three clinical questions. First, how common is occult endometrial carcinoma [16], and does its incidence vary across surgical populations and techniques? Second, does the surgical approach, particularly power morcellation or a laparoscopic route [17], influence prognosis when occult endometrial carcinoma is inadvertently treated as benign disease? Third, how should incidentally diagnosed endometrial carcinoma be managed [18], given that the initial operation was not performed as a cancer-directed staging procedure?

In this review, occult endometrial carcinoma was defined as endometrial carcinoma first diagnosed on final histopathological examination of a hysterectomy specimen, in a woman whose hysterectomy was performed for a presumed benign indication and who had no preoperative diagnosis or clinical suspicion of uterine malignancy. The terms “occult,” “incidental,” “unexpected,” “unsuspected,” and “unanticipated” are used variably in the literature. In this review, they are regarded as synonymous when they conform to this definition.

Although numerous single-institution series, national database analyses, and registry-linked cohorts have addressed these questions, their estimates vary widely, and no previous synthesis has comprehensively integrated the incidence, prognosis, and management of occult endometrial carcinoma [16,19]. Therefore, a systematic review and meta-analysis was conducted to (1) estimate the incidence of occult endometrial carcinoma after hysterectomy for benign disease, defined as the proportion of hysterectomies in which carcinoma is identified on final pathology, with pre-specified procedural subgroups; (2) evaluate the prognostic impact of morcellation and surgical route; and (3) summarize management strategies and outcomes following an incidental diagnosis.

## 2. Materials and Methods

### 2.1. Protocol and Search

The review followed the Preferred Reporting Items for Systematic Reviews and Meta-Analyses 2020 guidelines (Supplementary Table S1), and the protocol was registered retrospectively in the Open Science Framework (DOI: 10.17605/OSF.IO/NA8F9). PubMed, Scopus, and the Cochrane Central Register of Controlled Trials were searched from inception to 30 September 2026 (date of the final search). Because a sensitivity check showed that an initial strategy containing an outcome block (prognosis, survival, recurrence, or re-staging) retrieved only 14 of the 29 studies identified at that time, the search was revised and re-run without the outcome block. The final strategy combined three concept blocks using the Boolean operator AND: (1) endometrial, uterine, or gynecologic malignancy, (2) hysterectomy, morcellation, or sacrocolpopexy, and (3) occult, incidental, unexpected, unsuspected, unanticipated, or undiagnosed diagnosis. The revised strategy retrieved all previously included studies. Reference lists of included studies and relevant systematic reviews were also hand-searched. Complete database-specific search strategies are provided in Supplementary Table S2.

### 2.2. Eligibility

Studies were eligible if they included women who underwent hysterectomy for a presumed benign indication and reported the incidence of occult endometrial or uterine malignancy and/or survival or management outcomes of occult endometrial carcinoma. Because the included studies described these cases with different terms (occult, incidental, unexpected, unsuspected, or unanticipated malignancy), eligibility was based on the case definition rather than on terminology. Cases had to be endometrial carcinomas first identified on final pathology, without a preoperative diagnosis or suspicion of malignancy. When a study reported mixed populations, only the benign-indication denominator and corresponding cases were extracted if these data were separable, and the terminology and case definition used by each study were recorded. Studies were excluded if they involved surgery for known or suspected malignancy, did not report a uterine or oncologic outcome, or otherwise did not meet the predefined population/patient, intervention, comparison, and outcome criteria. Hysteroscopic surgery for submucosal myoma or polyps was also excluded because it does not involve hysterectomy. Conference abstracts, letters, and case reports were excluded, and when studies reported overlapping populations, the larger or more complete report was retained.

### 2.3. Screening and Data Extraction

Two reviewers (YHC and DCD) independently performed study screening and data extraction. After deduplication in Rayyan (Rayyan Systems, Inc., Cambridge, MA, USA), records were screened by title and abstract, followed by full-text assessment of potentially eligible studies. Data were extracted in two stages and included study design, country, population characteristics, denominator, occult malignancy, endometrial carcinoma events, stage and grade distribution, adjusted hazard ratios (HRs) for survival, and management outcomes (re-staging, upstaging, lymphadenectomy, and adjuvant therapy).

### 2.4. Risk of Bias Assessment

Risk of bias was assessed with design-specific tools. Studies contributing incidence data (*n* = 72) were appraised with the Joanna Briggs Institute (JBI) Critical Appraisal Checklist for Studies Reporting Prevalence Data, which comprises nine items [20]; sample size was considered adequate if at least 1277 hysterectomies were included, corresponding to a precision of ±0.3 percentage points at an expected incidence of 0.3%, and studies meeting ≥7, 4–6, or ≤3 items were rated as having low, moderate, or high risk of bias, respectively. Studies contributing prognostic or management data (*n* = 8) were assessed with the Quality In Prognosis Studies (QUIPS) tool across six domains: study participation (D1), study attrition (D2), prognostic factor or exposure measurement (D3), outcome measurement (D4), study confounding and adjustment (D5), and statistical analysis and reporting (D6). Each domain was rated as having low, moderate, or high risk of bias, with an overall risk-of-bias judgment assigned to each study [21]. QUIPS assessments of the original studies were performed independently by two reviewers (YHC and DCD). For the JBI appraisal and for studies added in the updated search, initial ratings were drafted with the assistance of an artificial intelligence tool (Opus 5.5, Claude, Anthropic) and independently assessed by DCD. Disagreements were resolved by discussion until a consensus was reached. Inter-rater agreement was quantified using percentage agreement and Cohen’s κ. JBI results are summarized by item and overall judgment, and QUIPS results are presented as a traffic-light plot with a domain-level summary; study-level ratings are provided in Supplementary Table S3.

### 2.5. Quantitative Synthesis

Analyses were performed using Python 3.14.7 (NumPy 2.5.4/Matplotlib 3.11.2), with independent verification of the results. The incidence of occult endometrial carcinoma was pooled using a random-effects DerSimonian–Laird meta-analysis of proportions on the Freeman–Tukey double-arcsine scale and back-transformed to percentages. Throughout this review, “incidence” denotes the proportion of hysterectomies performed for presumed benign indications in which occult carcinoma was identified on final histopathology (number of cases divided by number of hysterectomies). Because ascertainment occurs at a single time point (surgery), this measure corresponds to a period prevalence or cumulative proportion at hysterectomy rather than an incidence rate based on person-time; the term is retained for consistency with the primary literature. Per-study Wilson 95% confidence intervals (CIs) were reported. Pre-specified subgroups included all benign hysterectomies, morcellation-specific procedures, and hysterectomies performed for POP. Because individual patient data were unavailable, subgroups were assigned at the study level according to the source population defined by each study, and each study contributed to only one subgroup. A hierarchical rule was applied: studies restricted to hysterectomy for POP were assigned to the POP subgroup regardless of surgical technique; studies restricted to procedures involving morcellation for other benign indications were assigned to the morcellation-specific subgroup; and studies of unselected benign hysterectomies with mixed indications and techniques were assigned to the all-benign subgroup. The POP subgroup was therefore defined by indication and the morcellation-specific subgroup by surgical technique. A sensitivity analysis reassigned POP cohorts in which all specimens were morcellated to the morcellation-specific subgroup. A secondary analysis pooled all occult uterine malignancies, including endometrial carcinomas and sarcomas. Studies that reported uterine corpus malignancy without separating endometrial carcinoma from sarcoma were included only in this secondary analysis. 95% prediction intervals were calculated to estimate the expected range of incidence in a new comparable setting. Potential sources of heterogeneity were explored using subgroup tests for differences; random-effects meta-regression (method of moments) for publication year, sample size (log-transformed), data source (administrative or registry database versus institutional cohort), and geographic region; and a leave-one-out sensitivity analysis. Statistical heterogeneity was assessed using I^2^, with values of ≤25%, ≥50%, and ≥75% indicating low, moderate, and high heterogeneity, respectively. Additional sensitivity analyses excluded a study with partially overlapping enrolment and studies with atypical source populations. Sensitivity analyses also used alternative random-effects approaches suited to rare proportions: a binomial–normal generalized linear mixed model (random-intercept logistic regression) fitted by maximum likelihood with adaptive Gauss–Hermite quadrature, which does not require continuity correction for zero-event studies; restricted maximum likelihood and Paule–Mandel estimators of between-study variance; the Hartung–Knapp–Sidik–Jonkman adjustment; and logit-transformed proportions. Subgroup differences were assessed formally using the test for subgroup differences, a likelihood-ratio test in the generalized linear mixed model, and random-effects meta-regression. Survival and management outcomes were synthesized narratively as the exposures and effect measures were not sufficiently comparable for quantitative pooling.

## 3. Results

### 3.1. Study Selection

**Figure 1:**
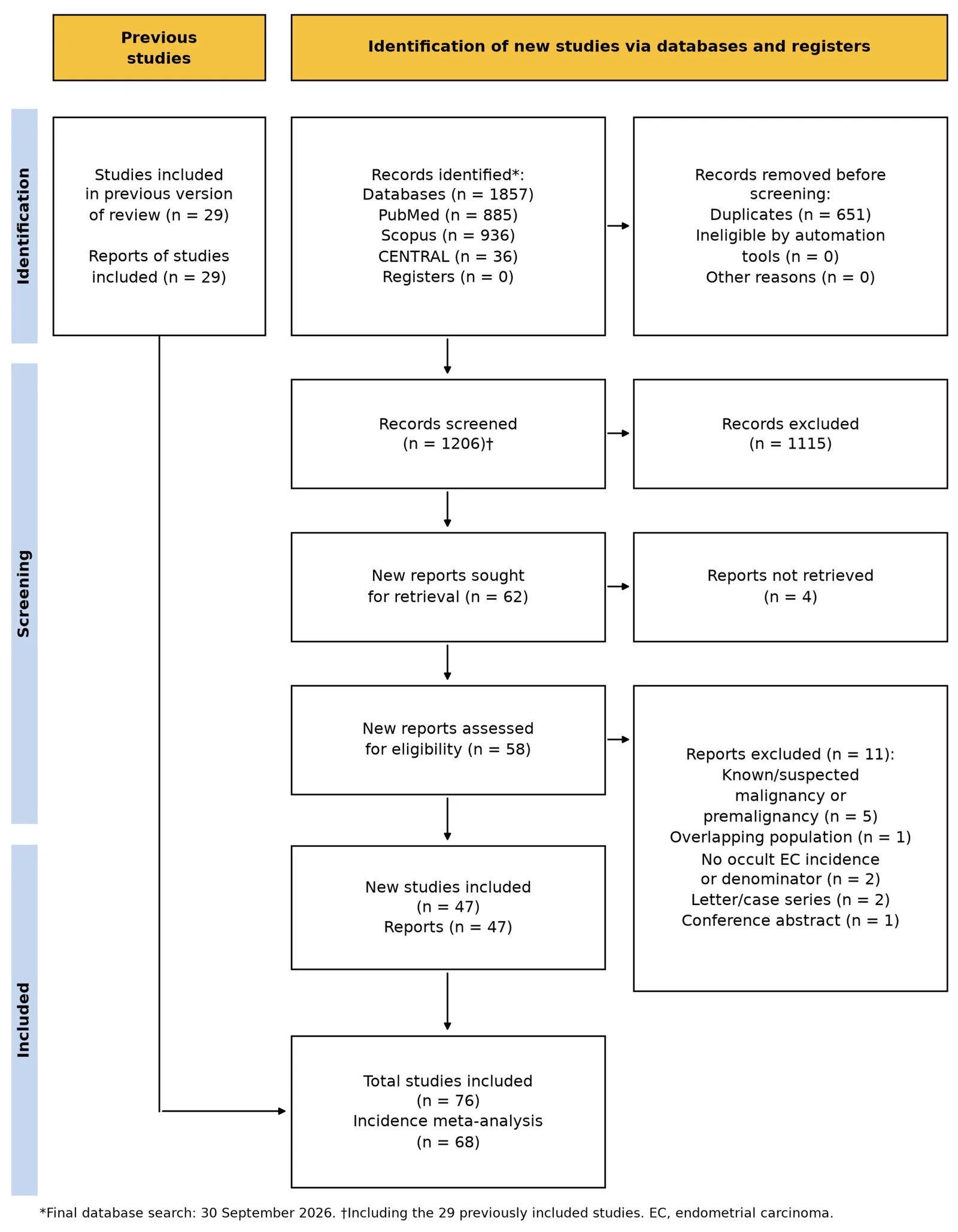
Preferred Reporting Items for Systematic Reviews and Meta-Analyses 2020 flow diagram.

The updated database search identified 1857 records (PubMed: 885; Scopus: 936; CENTRAL: 36). After removing 651 duplicates, 1206 records were screened by title and abstract; all 29 studies included in the previous version of this review were retrieved using the revised strategy, and 1115 records were excluded. Of 62 new reports sought for retrieval, 4 could not be obtained. Of the 58 full-text reports assessed, 11 were excluded: 5 because the study population included women with known or suspected malignancy or premalignancy, 1 because of an overlapping population, 2 because occult endometrial carcinoma incidence or an appropriate denominator could not be extracted, 2 letters or case series, and 1 conference abstract. Thus, 47 new studies were added to the 29 previously included studies, yielding 76 studies (Figure 1). The included studies comprised single-institution and multicenter cohorts, large national and administrative database analyses [22,23,24,25], and registry-linked survival studies [9], published from 1970 to 2026 across 22 countries and regions. Study characteristics are summarized in Table 1.

### 3.2. Incidence of Occult Endometrial Carcinoma

Sixty-eight studies provided extractable endometrial carcinoma–specific numerators and denominators; one national database study that reported only uterine corpus malignancy [22] contributed to the secondary analysis only.

**Figure 2:**
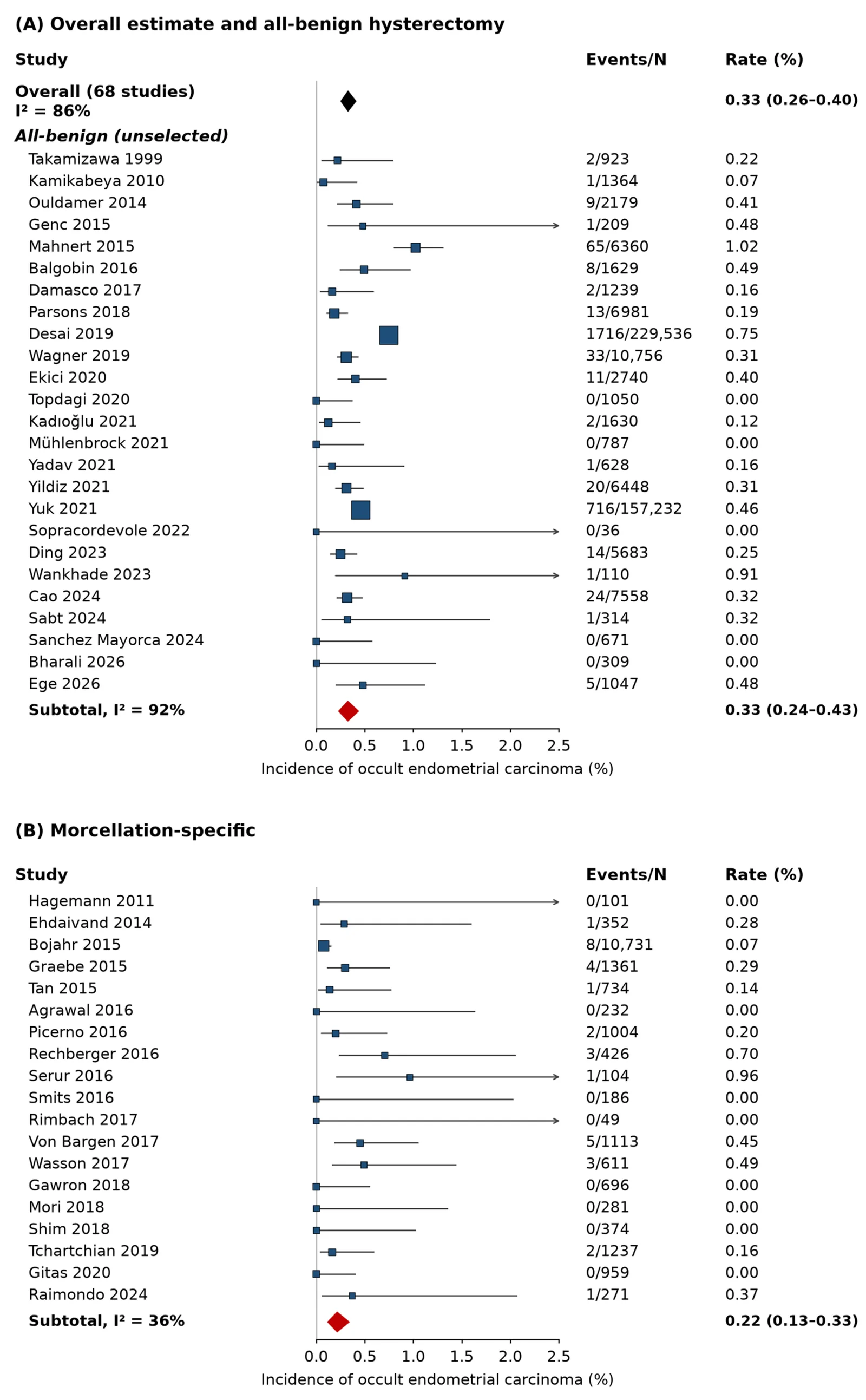

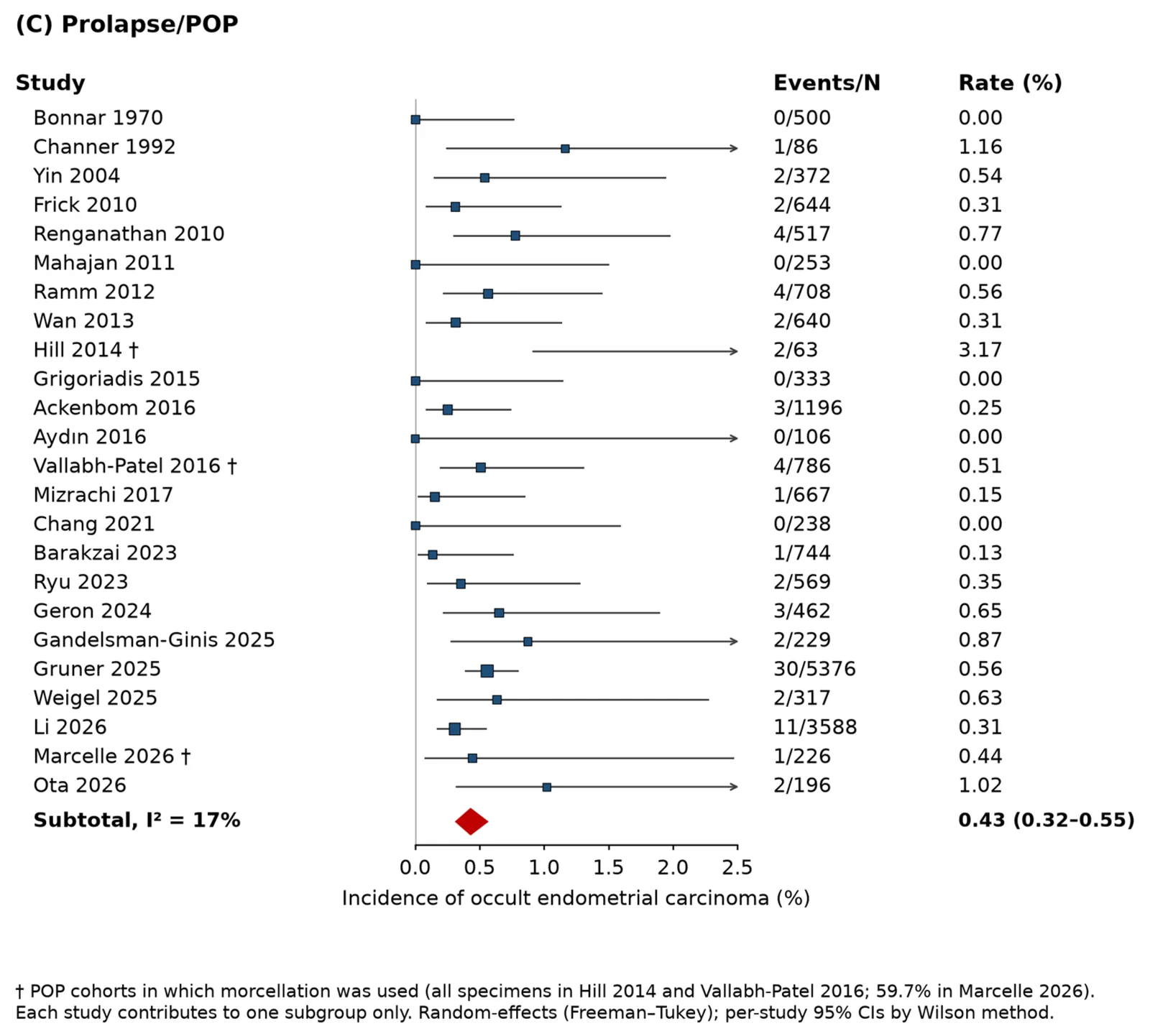
Pooled incidence of occult endometrial carcinoma after hysterectomy for benign disease, with pre-specified procedural subgroups. Overall pooled incidence, 0.33% (95% CI: 0.26–0.40). Each study contributes to one subgroup only; † indicates POP cohorts in which morcellation was used. (**A**) Overall estimate and all-benign hysterectomy subgroup [12,16,19,24,25,26,30,38,43,44,47,48,49,50,51,52,53,54,55,56,57,58,59,60,61]; (**B**) morcellation-specific subgroup [17,28,29,31,32,33,39,62,63,64,65,66,67,68,69,70,71,72,73]; (**C**) prolapse/POP subgroup [36,37,40,41,45,46,74,75,76,77,78,79,80,81,82,83,84,85,86,87,88,89,90,91]. Blue color: Rate of each study. Red color: Rate of meta-analysis.

The pooled incidence of occult endometrial carcinoma after hysterectomy for benign disease was 0.33% (95% CI: 0.26–0.40, I^2^ = 86%) (Figure 2 and Table 2). The subgroup estimates were as follows:

- All-benign hysterectomy, 25 studies [12,16,19,24,25,26,30,38,43,44,47,48,49,50,51,52,53,54,55,56,57,58,59,60,61]: 0.33% (95% CI: 0.24–0.43, I^2^ = 92%)
- Morcellation-specific, 19 studies [17,28,29,31,32,33,39,62,63,64,65,66,67,68,69,70,71,72,73]: 0.22% (95% CI: 0.13–0.33, I^2^ = 36%)
- Prolapse/POP, 24 studies [36,37,40,41,45,46,74,75,76,77,78,79,80,81,82,83,84,85,86,87,88,89,90,91]: 0.43% (95% CI: 0.32–0.55, I^2^ = 17%)

**Figure 3:**
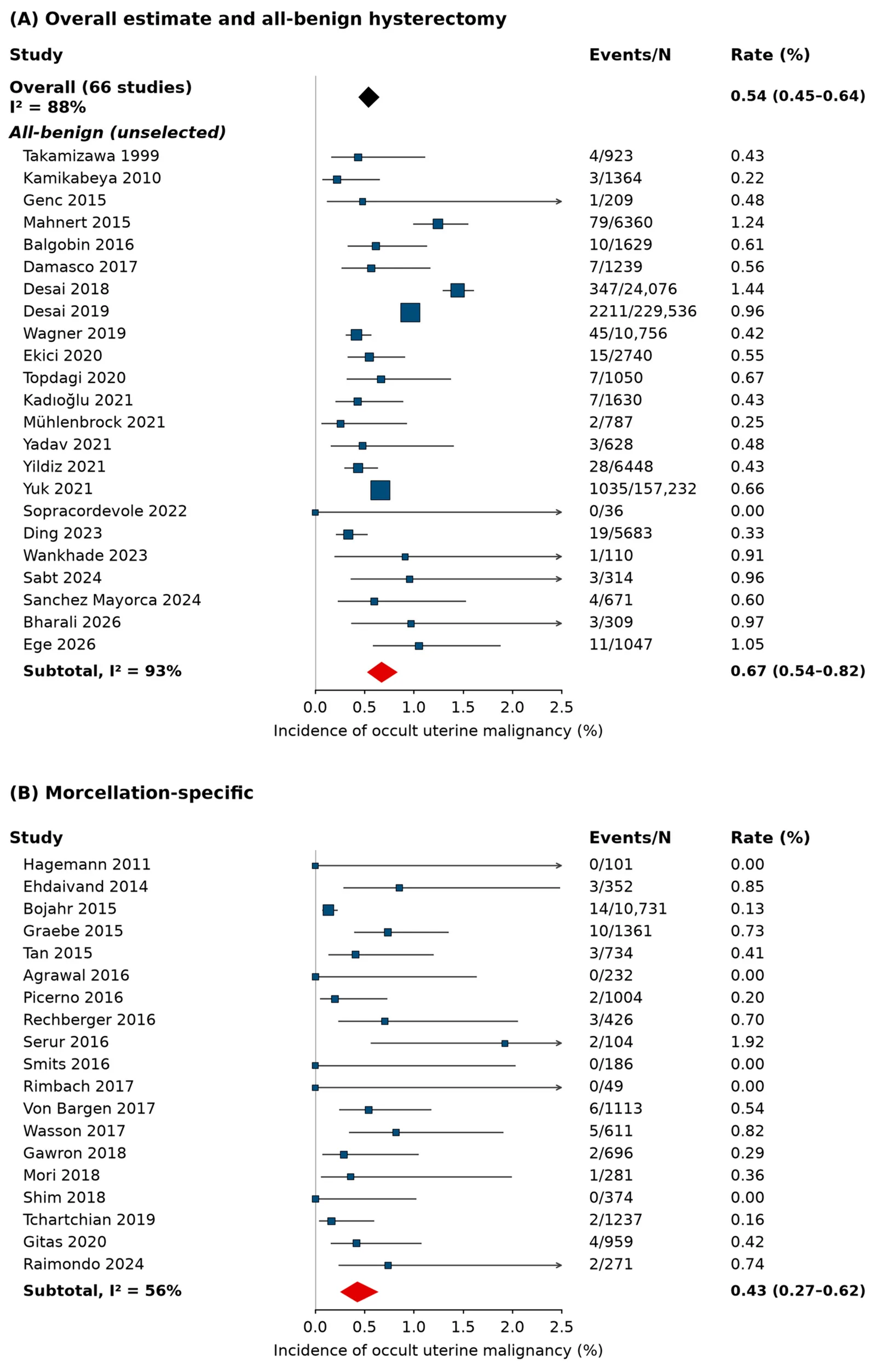

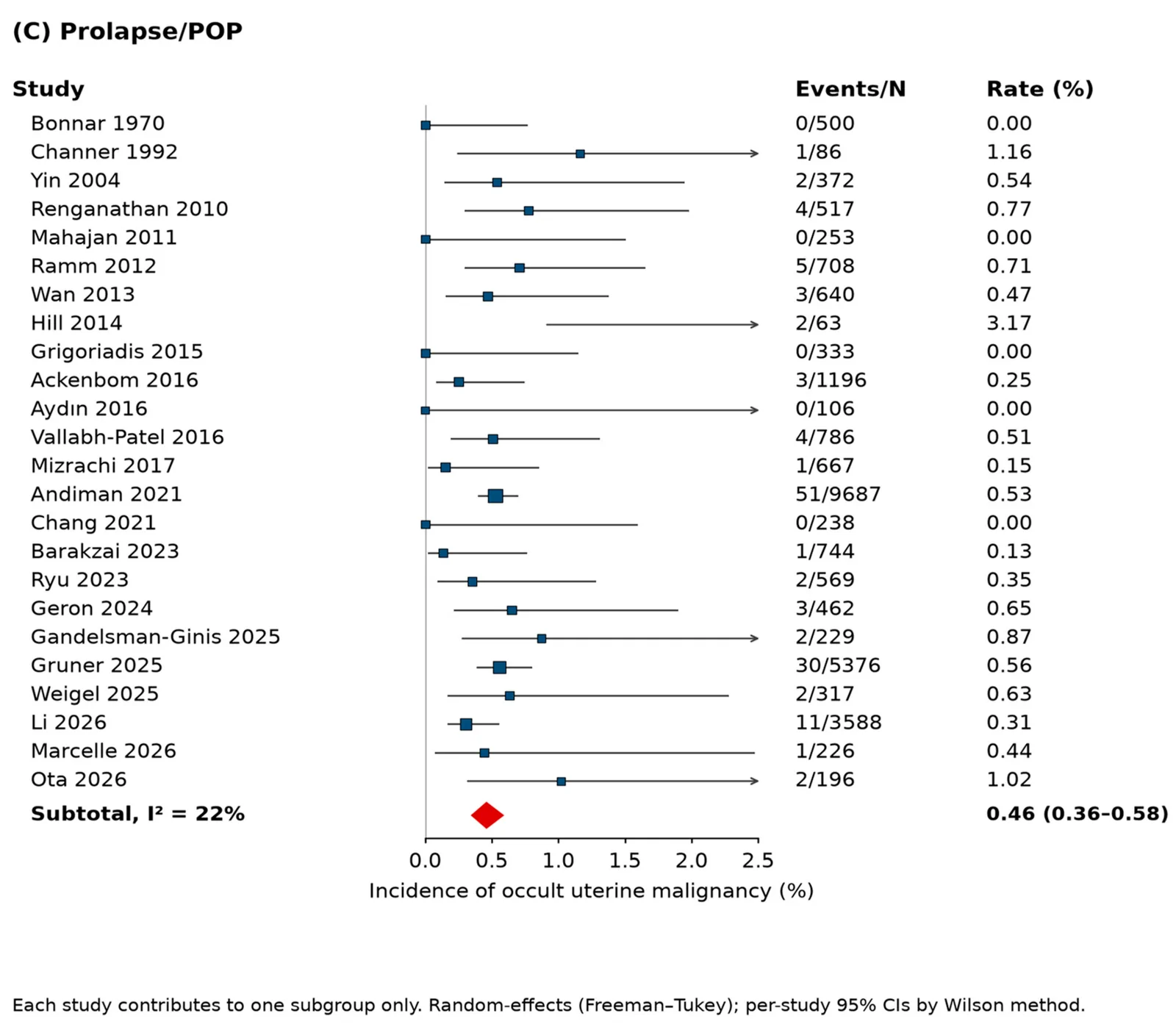
Pooled incidence of occult uterine malignancy, including endometrial carcinoma plus sarcoma. Overall pooled incidence, 0.54% (95% CI: 0.45–0.64). (**A**) Overall estimate and all-benign hysterectomy subgroup [12,22,24,25,26,30,38,44,47,48,49,50,51,52,53,54,55,56,57,58,59,60,61]; (**B**) morcellation-specific subgroup [17,28,29,31,32,33,39,62,63,64,65,66,67,68,69,70,71,72,73]; (**C**) prolapse/POP subgroup [23,36,37,41,45,46,74,75,76,77,78,79,80,81,82,83,84,85,86,87,88,89,90,91]. Blue color: Rate of each study. Red color: Rate of meta-analysis.

Each study contributed to only one subgroup. In a sensitivity analysis, when the two POP cohorts in which all specimens were morcellated [41,45] were reassigned to the morcellation-specific subgroup, the pooled incidence was 0.26% (95% CI: 0.15–0.39, I^2^ = 48%) for morcellation-specific cohorts and 0.42% (95% CI: 0.32–0.53, I^2^ = 9%) for POP cohorts, and the relative ordering of subgroups was unchanged. Excluding a study with enrolment partially overlapping that of another cohort from the same center [71] yielded 0.33% (95% CI: 0.26–0.41), and excluding three studies with atypical source populations [49,56,57] yielded 0.32% (95% CI: 0.26–0.40). The pooled estimate was robust to the choice of random-effects model: incidence ranged from 0.28% (95% CI: 0.22–0.35; binomial–normal generalized linear mixed model) to 0.37% (95% CI: 0.31–0.44; logit transformation), and estimates using restricted maximum likelihood, Paule–Mandel, and Hartung–Knapp–Sidik–Jonkman methods were 0.33% (Supplementary Table S4). Subgroup differences were supported by the test for subgroup differences (*p* = 0.03) and by a likelihood-ratio test in the generalized linear mixed model (*p* = 0.03), but not significantly by meta-regression with a common between-study variance (*p* = 0.08). The lower incidence in the morcellation subgroup is compatible with preoperative selection of patients considered suitable for morcellation, but it may equally reflect differences in age, indication, study population, preoperative screening practices, or study era, which could not be separated using study-level data. Incidence increased substantially with age, with both Desai (2018; uterine corpus malignancy) and Mahnert (2015) reporting higher rates among women aged ≥55 years [22,30]. In a secondary analysis, the pooled incidence of occult uterine malignancies, including sarcoma, across 66 studies was 0.54% (95% CI: 0.45–0.64, I^2^ = 88%) (Figure 3 and Table 2).

Heterogeneity was substantial (I^2^ = 86% overall), reflecting variation in denominators (36–229,536), study populations, and study periods, and the 95% prediction interval for the overall estimate was wide (0.04–0.87%), indicating that incidence in an individual setting may differ considerably from the pooled value. Prediction intervals were 0.05–0.85% for all-benign, 0.03–0.59% for morcellation-specific, and 0.21–0.73% for POP cohorts, and 0.13–1.22% for all occult uterine malignancy (Table 2). Surgical context explained part of the heterogeneity (test for subgroup differences, *p* = 0.03; approximately 22% of between-study variance), with low heterogeneity among POP (I^2^ = 17%) and morcellation-specific (I^2^ = 36%) cohorts. Incidence was higher in the three administrative or registry database studies than in institutional cohorts (*p* < 0.001) and varied by geographic region (*p* = 0.001), whereas publication year (*p* = 0.55) and sample size (*p* = 0.29) were not associated with incidence. In leave-one-out analysis, pooled estimates ranged from 0.31% to 0.34%; excluding the three database studies yielded 0.29% (95% CI: 0.24–0.35, I^2^ = 45%). An additional hand-searched study, a 2026 database analysis of supracervical hysterectomy, reported occult malignancy but did not separately report endometrial carcinoma; therefore, its findings were incorporated narratively [42].

### 3.3. Prognostic Impact of Morcellation and Surgical Route

Survival and management outcomes are summarized in Table 3. Two large studies reported adjusted survival estimates specific to endometrial carcinoma. In a New York registry–linked cohort of 843 patients with occult endometrial carcinoma, uncontained power morcellation during laparoscopic supracervical hysterectomy or myomectomy, compared with abdominal hysterectomy, was not significantly associated with disease-specific mortality (adjusted HR: 2.26, 95% CI: 0.87–5.88) [9]. This wide confidence interval is compatible both with no difference and with a more than twofold increase in mortality. Similarly, a Korean national cohort found no significant difference in 7-year overall survival between laparoscopic and laparotomic hysterectomy among patients with unsuspected endometrial carcinoma (HR: 0.76, 95% CI: 0.49–1.16) [24]. By contrast, both studies reported significant associations between morcellation or surgical route and survival in uterine sarcoma [9,24]. Uncontained morcellation was associated with higher disease-specific mortality in patients with occult sarcoma (adjusted HR: 2.66, 95% CI: 1.11–6.37), whereas laparoscopy was associated with lower mortality for non-endometrial malignancies (HR: 0.46, 95% CI: 0.26–0.80), although the latter finding was potentially confounded by differences in case mix. A large morcellation series reported no recurrences among 13 patients with occult malignancy during a median follow-up of 65.6 months [28]. These estimates were not pooled because the exposure comparisons differed (morcellation versus open surgery and laparoscopy versus open surgery), and the directions of the effect estimates also differed.

### 3.4. Stage, Grade, and Prognosis of Incidental Endometrial Carcinoma

Incidental endometrial carcinomas were predominantly early stage and low grade. Wagner et al. (2019) reported International Federation of Gynecology and Obstetrics stage IA in 81.8% of patients with occult endometrial cancers (Table 3) [12]. In a comparison of occult and preoperatively diagnosed endometrial carcinomas, Gruner et al. (2025) [36] found that occult tumors were more frequently grade I and had less lymphovascular space invasion, smaller tumor size, and less deep myometrial invasion. Five-year overall survival was 90.9% among patients with occult tumors versus 83.0% among those with preoperatively diagnosed tumors, whereas 5-year recurrence-free survival was 95.0% vs. 66.8%, respectively [36].

### 3.5. Management After Incidental Diagnosis

Evidence on management after incidental diagnosis was limited to small observational series. In a series of 20 patients with incidentally diagnosed endometrial carcinoma selected for high-risk features, completion staging upstaged 5 patients (25%) and informed adjuvant treatment decisions. Ultimately, 45% required no adjuvant therapy [34]. Because this series included only 20 patients selected for high-risk features, its upstaging rate cannot be generalized to unselected patients with low-risk disease. In a referral series of 17 patients with uterine malignancy diagnosed after supracervical hysterectomy or morcellation for presumed benign disease, including 8 with endometrioid carcinoma, 2 of 12 patients who underwent completion surgery were upstaged, both with leiomyosarcoma [92]. Evidence on the therapeutic value of nodal assessment was limited and imprecise. In a cohort undergoing complementary lymphadenectomy during a second surgery, 25% of patients had nodal involvement, but complementary lymphadenectomy was not independently associated with overall survival (HR: 0.40, 95% CI: 0.16–1.00, *p* = 0.051) or disease-free survival (HR: 0.77, 95% CI: 0.38–1.59); however, the confidence interval for overall survival was compatible with a substantial benefit, and the analysis was observational and subject to selection bias [35].

### 3.6. Risk of Bias

**Figure 4:**
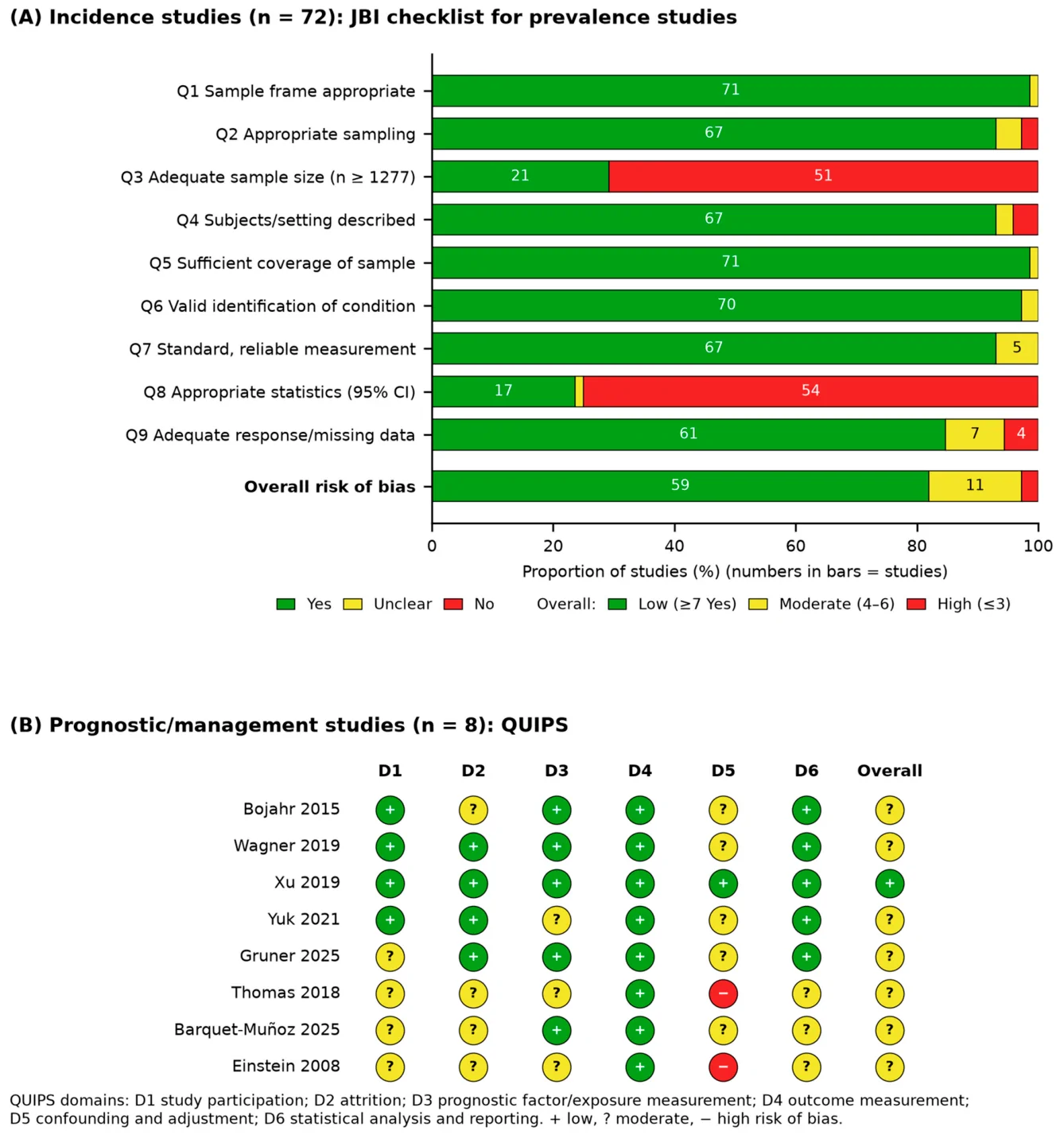
Risk of bias. (**A**) JBI Critical Appraisal Checklist for Studies Reporting Prevalence Data [20] in the 72 studies contributing incidence data; (**B**) QUIPS assessment [21] of the 8 studies contributing prognostic or management data: Bojahr 2015 [28], Wagner 2019 [12], Xu 2019 [9], Yuk 2021 [24], Gruner 2025 [36], Thomas 2018 [34], Barquet-Muñoz 2025 [35], and Einstein 2008 [92].

Risk-of-bias results are summarized in Figure 4 and Supplementary Table S3. For the JBI appraisal, inter-rater agreement was 99.8% (647 of 648 item ratings; Cohen’s κ = 0.996), and the single disagreement was resolved by consensus. Of the 72 incidence studies, 59 were rated as having a low, 11 a moderate, and 2 a high risk of bias. The sample frame (71 studies), coverage of the identified sample (71), and identification of the condition by histopathology or registry linkage (70) were appropriate in nearly all studies. The most frequently unmet items were an adequate sample size (met in 21 of 72 studies) and appropriate statistical analysis, as most studies reported incidence without a 95% CI (met in 17 of 72). Among the eight prognostic or management studies assessed with QUIPS (inter-rater agreement in the original assessment 98.9%, κ = 0.98), one was rated as having a low and seven a moderate overall risk of bias; outcome measurement was at low risk in all eight, whereas confounding and adjustment was the weakest domain (high risk in two and moderate in five studies). Overall, the incidence evidence was at predominantly low risk of bias, although its precision was limited by small single-centre series, and the prognostic evidence was at moderate risk of bias, driven mainly by residual confounding.

## 4. Discussion

This systematic review integrated evidence on the incidence, prognosis, and management of occult endometrial carcinoma diagnosed after hysterectomy for presumed benign diseases. Three findings are noteworthy.

First, occult endometrial carcinoma was uncommon, with a pooled incidence of 0.33%. However, given substantial heterogeneity, this figure should be regarded as an approximate summary rather than a precise estimate, and incidence in a given setting may plausibly range from below 0.1% to approximately 0.9% depending on patient age, selection, and surgical context. Incidence was lowest (0.2%) in cohorts preselected for morcellation and highest (0.4%) in prolapse cohorts. This difference was modest, was supported by formal subgroup testing in two of three analyses, and may reflect differences in patient selection, age, indication, screening practices, or study era rather than an effect of the procedure itself; these factors could not be disentangled with study-level data. Incidence increased substantially with age, with rates several-fold higher among women aged ≥55 years [22,30]. These findings identify older women as a population in whom preoperative endometrial evaluation and counseling may be particularly valuable, especially before minimally invasive or morcellation procedures and prolapse surgery.

Preoperative identification of occult endometrial carcinoma remains challenging, as many cases occurred after an unremarkable evaluation [19,79,83,87]. Transvaginal ultrasonography is recommended as the first-line evaluation of postmenopausal bleeding [93], whereas the optimal endometrial thickness threshold in asymptomatic women remains uncertain [94]; office endometrial sampling, with hysteroscopy-directed biopsy when needed, provides accurate tissue diagnosis in symptomatic women [95]. However, universal preoperative biopsy has yielded inconsistent results in benign hysterectomy cohorts [45,79]. A targeted approach appears more efficient: carcinoma was rare when cytology and ultrasonography were normal, and bleeding was absent [80], whereas risk increased with age and postmenopausal bleeding, and risk models achieved a negative predictive value of 99.8% [25]. DNA-methylation tests of cervicovaginal samples are promising [96,97]. Preoperative evaluation should therefore be tailored to risk, particularly in postmenopausal women and before morcellation, supracervical hysterectomy, or uterus-preserving prolapse surgery [14].

Second, the limited observational evidence available did not demonstrate a statistically significant association between morcellation or laparoscopic surgery and poorer survival in occult endometrial carcinoma. However, this evidence derives from only two studies reporting adjusted estimates, and the confidence intervals were wide; notably, the point estimate for uncontained power morcellation (adjusted HR 2.26, 95% CI 0.87–5.88) was compatible with more than a doubling of disease-specific mortality [9]. Residual confounding is also likely, because morcellation and surgical route were not randomly assigned and may be related to uterine size, patient age, and tumor characteristics. These data therefore preclude conclusions regarding the oncologic safety of morcellation or laparoscopic surgery in this setting. The contrast with uterine sarcoma, for which morcellation has been associated with increased recurrence and mortality [10], is biologically plausible, as low-grade, early-stage endometrioid carcinomas are generally less aggressive than sarcomas when disrupted. Nevertheless, most evidence of morcellation-related harm derives from occult leiomyosarcoma [11], and the absence of a demonstrated association for endometrial carcinoma should not be interpreted as evidence of no harm. Careful tissue extraction, including contained morcellation, remains warranted whenever occult malignancy cannot be excluded [98].

Third, incidental endometrial carcinoma was typically early-stage and low-grade, with a favorable prognosis and a 5-year overall survival of approximately 90% [36]. Management should therefore be individualized rather than reflexive. In a small series of 20 patients selected for high-risk features, completion staging upstaged one-quarter and could both intensify and de-escalate adjuvant therapy; this rate is unlikely to apply to unselected patients with low-risk disease [34]. Complementary lymphadenectomy provided additional staging information, but a single observational cohort did not demonstrate a statistically significant survival benefit (overall survival HR 0.40, 95% CI 0.16–1.00), an imprecise estimate that neither confirms nor excludes a benefit [35]. A risk-stratified approach that reserves re-staging for patients whose histopathologic features indicate a meaningful probability of extrauterine disease, consistent with contemporary molecularly integrated risk stratification, appears reasonable, although the supporting evidence is limited to small observational studies and should not be generalized to all patients with occult endometrial carcinoma [99].

### 4.1. Limitations

The incidence meta-analysis showed substantial heterogeneity, reflecting wide variation in denominators, study populations, study periods, and case definitions. Heterogeneity was only partially explained by surgical context and data source; age, which is likely a major contributor, could not be modeled because age-stratified data were reported inconsistently. The pooled estimates should therefore be interpreted as approximate summaries, with prediction intervals indicating the plausible range across settings. Subgroups were assigned at the study level and combined an indication-based category (POP) with a technique-based category (morcellation); unselected cohorts in the all-benign subgroup included some patients undergoing POP surgery or morcellation who could not be separated. All included studies were observational and predominantly retrospective. Four potentially eligible reports could not be retrieved, and conference abstracts were excluded. Two eligible studies could not contribute to the pooled estimate of occult endometrial carcinoma because relevant full-text data were unavailable. Theben (2013), a laparoscopic supracervical hysterectomy cohort involving morcellation, did not report the number of malignancies [27], whereas Le Neveu (2026), a large supracervical hysterectomy database study, did not allow occult malignancy total to be resolved to the endometrial carcinoma subset [42]; both studies were therefore incorporated narratively. The morcellation subgroup included both power and cold-knife or vaginal morcellation. One database study reported uterine rather than endometrium-specific malignancies and therefore contributed only to the secondary analysis. Survival evidence for occult endometrial carcinoma was limited to two observational studies with differing exposure definitions, imprecise estimates, and potential residual confounding, and could not be pooled; the absence of a statistically significant association therefore does not establish equivalence or the absence of harm. The pooled incidence represents the proportion of hysterectomies with occult carcinoma at a single time point rather than a person-time incidence rate, and it should not be interpreted as the population incidence of endometrial carcinoma. Management evidence was derived from small, selected observational series (20 and 17 patients) and a single cohort evaluating lymphadenectomy, which limits its generalizability.

Case definitions varied across the included studies, including in preoperative endometrial evaluation, the inclusion of myomectomy in some database cohorts, and whether the absence of preoperative suspicion could be confirmed, which probably added to the heterogeneity despite our use of a common operational definition. Finally, publication and referral patterns may have influenced incidence estimates from single-institution studies.

### 4.2. Future Directions

Prospective, age-stratified estimates of occult endometrial carcinoma before minimally invasive and prolapse surgery would improve risk assessment. Endometrial carcinoma-specific comparisons of contained versus uncontained tissue extraction and prospective evaluation of risk-stratified re-staging would inform clinical management.

## 5. Conclusions

Occult endometrial carcinoma occurs in approximately 0.2–0.4%, with a pooled incidence of 0.33%, and incidence increasing with age. The incidence was lowest among selected morcellation candidates (approximately 0.2%). The limited observational evidence available did not demonstrate a statistically significant association between morcellation or laparoscopic surgery and poorer survival in occult endometrial carcinoma. However, wide confidence intervals and potential residual confounding preclude conclusions regarding oncologic safety. Prognosis was generally favorable. These findings support age-informed preoperative endometrial evaluation and counseling and an individualized, risk-stratified approach to completion staging after incidental diagnosis, although evidence on management remains limited to small observational studies.

## Figures and Tables

**Table 1:** Characteristics of the 76 included studies.

#	Study	Year	Country	Design	Population (N)	Primary Contribution
1	Kamikabeya et al. [26]	2010	Brazil	Retrospective	1364 (leiomyoma)	Incidence
2	Theben et al. [27]	2013	Germany	Retrospective	1584 (LASH)	Incidence
3	Bojahr et al. [28]	2015	Germany	Retrospective	10,731 (LASH morcellation)	Incidence + prognosis
4	Graebe et al. [29]	2015	USA	Retrospective cohort	1361 (MIS morcellation)	Incidence
5	Mahnert et al. [30]	2015	USA	Database (MSQC)	6360 (benign hysterectomy)	Incidence
6	Tan et al. [31]	2015	Australia	Retrospective	734 (morcellation)	Incidence
7	Picerno et al. [32]	2016	USA	Retrospective cohort	1004 (power morcellation)	Incidence
8	Wasson et al. [33]	2017	USA	Retrospective cohort	611 morcellated (of 2296)	Incidence
9	Desai et al. [22]	2018	USA	Database (NSQIP)	24,076 (benign hysterectomy)	Prevalence
10	Thomas et al. [34]	2018	India	Case series	20 (incidental EMCA)	Management/re-staging
11	Wagner et al. [12]	2019	Germany	Retrospective	10,756 (benign hysterectomy)	Incidence + outcome
12	Xu et al. [9]	2019	USA	Registry-linked cohort	843 occult EMCA	Prognosis (mortality)
13	Andiman et al. [23]	2021	USA	Database (NSQIP)	9687 (POP hysterectomy)	Prevalence
14	Yuk et al. [24]	2021	Korea	National claims cohort	157,232 (benign hysterectomy)	Incidence + survival
15	Barquet-Muñoz et al. [35]	2025	Mexico	Retrospective cohort	260 (incidental EMCA)	Management (lymphadenectomy)
16	Gruner et al. [36]	2025	USA	Retrospective cohort	5376 (POP surgery)	Incidence + occult vs. preop
17	Marcelle et al. [37]	2026	Belgium	Retrospective	226 (sacrocolpopexy + SCH)	Incidence
18	Ding et al. [38]	2023	China	Retrospective	5683 (benign hysterectomy)	Incidence (14 EMCA)
19	Ehdaivand et al. [39]	2014	USA	Retrospective (morcellation)	352 (LHM)	Incidence (1 EMCA)
20	Frick et al. [40]	2010	USA	Retrospective (POP)	644 (POP)	Incidence (2 EMCA)
21	Gawron et al. [17]	2018	Poland	Retrospective (LSH)	696 (morcellation)	Incidence (0 EMCA)
22	Hill et al. [41]	2014	USA	Retrospective (POP)	63 (robotic SCH)	Incidence (2 EMCA)
23	Le Neveu et al. [42]	2026	USA	Database (Premier)	25,126 (supracervical hysterectomy)	Incidence
24	Ouldamer et al. [43]	2014	France	Retrospective	2179 (benign hysterectomy)	Incidence (9 EMCA)
25	Parsons et al. [16]	2018	USA	Retrospective	6981 (benign hysterectomy)	Incidence (13 EMCA)
26	Topdagi Yilmaz et al. [44]	2020	Turkey	Retrospective	1050 (benign hysterectomy)	Incidence (0 EMCA)
27	Vallabh-Patel et al. [45]	2016	USA	Retrospective (POP)	786 (sacrocolpopexy)	Incidence (4 EMCA)
28	Wan et al. [46]	2013	Hong Kong	Retrospective (POP)	640 (POP)	Incidence (2 EMCA)
29	Yildiz et al. [47]	2021	Turkey	Retrospective	6448 (benign hysterectomy)	Incidence (20 EMCA)
30	Takamizawa et al. [48]	1999	Japan	Retrospective	923 (leiomyoma)	Incidence (2 EMCA)
31	Genc et al. [49]	2015	Turkey	Retrospective	209 (postmenopausal, no PMB)	Incidence (1 EMCA)
32	Balgobin et al. [50]	2016	USA	Retrospective	1629 (VH/LAVH)	Incidence (8 EMCA)
33	Damasco et al. [51]	2017	Australia	Retrospective	1239 (myoma hysterectomy)	Incidence (2 EMCA)
34	Desai et al. [25]	2019	USA	Database (SPARCS)	229,536 (benign hysterectomy)	Incidence (1716 EMCA)
35	Ekici et al. [52]	2020	Turkey	Retrospective	2740 (benign hysterectomy)	Incidence (11 EMCA)
36	Kadıoğlu et al. [53]	2021	Turkey	Retrospective	1630 (benign hysterectomy)	Incidence (2 EMCA)
37	Mühlenbrock et al. [54]	2021	Chile	Retrospective	787 (myoma hysterectomy)	Incidence (0 EMCA)
38	Yadav et al. [55]	2021	India	Retrospective	628 (benign hysterectomy)	Incidence (1 EMCA)
39	Sopracordevole et al. [56]	2022	Italy	Retrospective	36 (cervical stenosis)	Incidence (0 EMCA)
40	Wankhade et al. [57]	2023	India	Retrospective	110 (benign hysterectomy)	Incidence (1 EMCA)
41	Cao et al. [19]	2024	China	Retrospective	7558 (benign hysterectomy)	Incidence (24 EMCA)
42	Sabt et al. [58]	2024	Bahrain	Retrospective	314 (benign hysterectomy)	Incidence (1 EMCA)
43	Sanchez Mayorca et al. [59]	2024	Colombia	Retrospective	671 (benign hysterectomy)	Incidence (0 EMCA)
44	Bharali et al. [60]	2026	India	Retrospective	309 (fibroid hysterectomy)	Incidence (0 EMCA)
45	Ege et al. [61]	2026	Turkey	Retrospective	1047 (benign hysterectomy)	Incidence (5 EMCA)
46	Hagemann et al. [62]	2011	USA	Retrospective (morcellation)	101 (morcellated hysterectomy)	Incidence (0 EMCA)
47	Agrawal et al. [63]	2016	India	Retrospective (morcellation)	232 (vaginal/laparoscopic morcellation)	Incidence (0 EMCA)
48	Rechberger et al. [64]	2016	Poland	Retrospective (morcellation)	426 (LASH morcellation)	Incidence (3 EMCA)
49	Serur et al. [65]	2016	USA	Retrospective (morcellation)	104 (contained manual morcellation)	Incidence (1 EMCA)
50	Smits et al. [66]	2016	Netherlands	Retrospective (morcellation)	186 (LSH morcellation)	Incidence (0 EMCA)
51	Rimbach et al. [67]	2017	Germany	Prospective cohort	49 (in-bag morcellation)	Incidence (0 EMCA)
52	Von Bargen et al. [68]	2017	USA	Retrospective (morcellation)	1113 (power morcellation)	Incidence (5 EMCA)
53	Mori et al. [69]	2018	USA	Retrospective (morcellation)	281 (contained power morcellation)	Incidence (0 EMCA)
54	Shim et al. [70]	2018	USA	Retrospective (morcellation)	374 (LSH morcellation)	Incidence (0 EMCA)
55	Tchartchian et al. [71]	2019	Germany	Retrospective (morcellation)	1237 (LASH/LAVH morcellation)	Incidence (2 EMCA)
56	Gitas et al. [72]	2020	Germany	Retrospective (morcellation)	959 (power morcellation)	Incidence (0 EMCA)
57	Raimondo et al. [73]	2024	Italy	Retrospective (morcellation)	271 (vaginal manual morcellation)	Incidence (1 EMCA)
58	Bonnar et al. [74]	1970	UK	Retrospective (POP)	500 (POP)	Incidence (0 EMCA)
59	Channer et al. [75]	1992	UK	Retrospective (POP)	86 (POP)	Incidence (1 EMCA)
60	Yin et al. [76]	2004	USA	Retrospective (POP)	372 (POP)	Incidence (2 EMCA)
61	Renganathan et al. [77]	2010	UK	Retrospective (POP)	517 (POP)	Incidence (4 EMCA)
62	Mahajan et al. [78]	2011	India	Retrospective (POP)	253 (POP)	Incidence (0 EMCA)
63	Ramm et al. [79]	2012	USA	Retrospective (POP/UI)	708 (POP/UI)	Incidence (4 EMCA)
64	Grigoriadis et al. [80]	2015	Greece	Retrospective (POP)	333 (POP)	Incidence (0 EMCA)
65	Ackenbom et al. [81]	2016	USA	Retrospective (POP)	1196 (POP)	Incidence (3 EMCA)
66	Aydin et al. [82]	2016	Turkey	Retrospective (POP)	106 (POP)	Incidence (0 EMCA)
67	Mizrachi et al. [83]	2017	Israel	Retrospective (POP)	667 (POP)	Incidence (1 EMCA)
68	Chang et al. [84]	2021	USA	Retrospective (POP)	238 (sacrocolpopexy + hysterectomy)	Incidence (0 EMCA)
69	Barakzai et al. [85]	2023	USA	Retrospective (POP)	744 (POP)	Incidence (1 EMCA)
70	Ryu et al. [86]	2023	Korea	Retrospective (POP)	569 (POP)	Incidence (2 EMCA)
71	Geron et al. [87]	2024	Israel	Retrospective (POP)	462 (POP)	Incidence (3 EMCA)
72	Gandelsman-Ginis et al. [88]	2025	USA	Retrospective (POP)	229 (POP)	Incidence (2 EMCA)
73	Weigel et al. [89]	2025	USA	Retrospective (POP)	317 (POP, postmenopausal)	Incidence (2 EMCA)
74	Li et al. [90]	2026	China	Retrospective (POP)	3588 (POP)	Incidence (11 EMCA)
75	Ota et al. [91]	2026	Japan	Retrospective (POP)	196 (sacrocolpopexy + hysterectomy)	Incidence (2 EMCA)
76	Einstein et al. [92]	2008	USA	Case series (referral)	17 (incidental uterine malignancy)	Management/re-staging

LASH, laparoscopy-assisted supracervical hysterectomy; MIS, minimally invasive surgery; emca, endometrial cancer; POP, pelvic organ prolapse; SCH, supracervical hysterectomy.

**Table 2:** Pooled incidence of occult endometrial carcinoma.

Subgroup	k	Pooled Incidence	95% CI	95% PI	I^2^
All-benign hysterectomy	25	0.33%	0.24–0.43	0.05–0.85	92%
Morcellation-specific	19	0.22%	0.13–0.33	0.03–0.59	36%
Prolapse/POP	24	0.43%	0.32–0.55	0.21–0.73	17%
Overall	68	0.33%	0.26–0.40	0.04–0.87	86%
Secondary: all uterine malignancy (EMCA + sarcoma)	66	0.54%	0.45–0.64	0.13–1.22	88%

POP: pelvic organ prolapse, emca: endometrial cancer, CI: confidence interval, PI: prediction interval. Incidence denotes the proportion of hysterectomies with occult carcinoma on final pathology.

**Table 3:** Survival and management outcomes.

Study	Outcome	Estimate
Xu 2019 [9]	Power morcellation vs. abdominal, disease-specific mortality (occult EMCA)	aHR 2.26 (0.87–5.88), NS
Xu 2019 [9]	Same, occult uterine sarcoma	aHR 2.66 (1.11–6.37), significant
Yuk 2021 [24]	Laparoscopy vs. laparotomy, 7-yr OS (unsuspected EMCA)	HR 0.76 (0.49–1.16), NS
Gruner 2025 [36]	5-yr OS: occult vs. preoperatively-diagnosed EMCA	90.9% vs. 83.0%
Gruner 2025 [36]	5-yr recurrence-free survival: occult vs. preop	95.0% vs. 66.8%
Wagner 2019 [12]	Stage distribution of occult EMCA	81.8% FIGO IA
Thomas 2018 [34]	Upstaging after completion staging	5/20 (25%)
Barquet-Muñoz 2025 [35]	Complementary lymphadenectomy, multivariate OS	HR 0.40 (0.16–1.00), *p* = 0.051 (NS)

Emca: endometrial cancer, os: overall survival, yr: years, pre-op: pre-operative, ns: not significant.

## Data Availability

All data analyzed in this review were derived from published studies and are presented in the article and its supplementary materials. The extracted datasets and analysis scripts are available from the corresponding author upon request.

## Supplementary Materials

The following supporting information can be downloaded at: https://www.mdpi.com/article/10.3390/diagnostics16213450/s1, Table S1: PRISMA 2020 checklist; Table S2: Database-specific search strategies; Table S3: Study-level risk-of-bias assessment; Table S4: Sensitivity of pooled incidence estimates to the random-effects model. Reference [100] is cited in the Supplementary Materials.

## Abbreviations

The following abbreviations are used in this manuscript:

CI	confidence interval
D1–D6	domains 1–6 of the Quality In Prognosis Studies tool
EMCA	endometrial carcinoma
FIGO	International Federation of Gynecology and Obstetrics
HR	hazard ratio
I^2^	I-squared statistic
JBI	Joanna Briggs Institute
LASH	laparoscopic-assisted supracervical hysterectomy
LHM	laparoscopic hysterectomy with morcellation
LSH	laparoscopic supracervical hysterectomy
MIS	minimally invasive surgery
NSQIP	National Surgical Quality Improvement Program
OSF	Open Science Framework
PICO	Population, Intervention, Comparison, and Outcome
POP	pelvic organ prolapse
PRISMA	Preferred Reporting Items for Systematic Reviews and Meta-Analyses
QUIPS	Quality In Prognosis Studies
SCH	supracervical hysterectomy
