# Supplementary material for: Incidence, Prognosis, and Management of Occult Endometrial Carcinoma After Hysterectomy for Benign Disease: A Systematic Review and Meta-Analysis
Source: Diagnostics (Basel). 2026 Oct 22;16(21):3450. doi: 10.3390/diagnostics16213450 (PMC13762430; doi:10.3390/diagnostics16213450)
Supplement: Supplementary file 1 [file diagnostics-16-03450-s001.zip › diagnostics-4553904-supplementary.pdf]

Supplementary Table S2. Database-specific search strategies

Searches were run from database inception to 30 September 2026 (date of the final search). No language, date, or study-design filters were applied. The strategies combine three concept blocks with AND: (1) endometrial/uterine or gynecologic malignancy, (2) hysterectomy or morcellation, and (3) occult/incidental/unexpected diagnosis. No outcome (prognosis/survival) block was used, so that incidence-only studies were not excluded.

PubMed (MEDLINE)

Line	Search
#1	"endometrial neoplasms"[MeSH] OR "uterine neoplasms"[MeSH] OR "corpus uteri"[MeSH] OR "endometrial cancer"[tiab] OR "endometrial carcinoma"[tiab] OR "endometrial adenocarcinoma"[tiab] OR "uterine cancer"[tiab] OR "uterine malignancy"[tiab] OR "uterine malignancies"[tiab] OR "uterine neoplasm*"[tiab] OR "uterine sarcoma*"[tiab] OR "gynecologic malignanc*"[tiab] OR "gynaecologic malignanc*"[tiab] OR "gynecological malignanc*"[tiab] OR "gynaecological malignanc*"[tiab] OR "gynecologic cancer*"[tiab] OR "gynaecologic cancer*"[tiab] OR "gynecological cancer*"[tiab] OR "gynaecological cancer*"[tiab] OR "occult malignanc*"[tiab]
#2	"hysterectomy"[MeSH] OR "hysterectom*"[tiab] OR "morcellation"[MeSH] OR "morcellat*"[tiab] OR "sacrocolpopexy"[tiab]
#3	"incidental findings"[MeSH] OR "occult"[tiab] OR "incidental*"[tiab] OR "unexpected"[tiab] OR "unsuspected"[tiab] OR "unanticipated"[tiab] OR "undiagnosed"[tiab] OR "inadequately staged"[tiab] OR "postoperative diagnosis"[tiab]
#4	#1 AND #2 AND #3

Records retrieved:885. Validation: this strategy retrieved all 29 studies included in the original review (entry date ≤ 11 June 2026: 871 records).

Scopus (Elsevier)

Line	Search
#1	TITLE-ABS-KEY("endometrial neoplasm*" OR "endometrial cancer*" OR "endometrial carcinoma*" OR "endometrial adenocarcinoma*" OR "uterine cancer*" OR "uterine malignanc*" OR "uterine neoplasm*" OR "uterine sarcoma*" OR "corpus uteri" OR "gynecologic malignanc*" OR "gynaecologic malignanc*" OR "gynecological malignanc*" OR "gynaecological malignanc*" OR "gynecologic cancer*" OR "gynaecologic cancer*" OR "gynecological cancer*" OR "gynaecological cancer*" OR "occult malignanc*")
#2	TITLE-ABS-KEY(hysterectom* OR morcellat* OR sacrocolpopexy)
#3	TITLE-ABS-KEY(occult OR incidental* OR unexpected OR unsuspected OR unanticipated OR undiagnosed OR "inadequately staged" OR "postoperative diagnosis")
#4	#1 AND #2 AND #3 (combined in Advanced search as: TITLE-ABS-KEY(…#1…) AND TITLE-ABS-KEY(…#2…) AND TITLE-ABS-KEY(…#3…))

Records retrieved:936.

Cochrane Central Register of Controlled Trials (CENTRAL), Cochrane Library

Line	Search
#1	MeSH descriptor: [Endometrial Neoplasms] explode all trees

#2	MeSH descriptor: [Uterine Neoplasms] explode all trees
#3	("endometrial cancer*" OR "endometrial carcinoma*" OR "endometrial adenocarcinoma*" OR "uterine cancer*" OR "corpus uteri" OR "occult malignanc*"):ti,ab,kw
#4	((uterine OR uterus OR gynecologic* OR gynaecologic*) NEXT (malignan* OR neoplasm* OR sarcoma* OR cancer*)):ti,ab,kw
#5	#1 OR #2 OR #3 OR #4
#6	MeSH descriptor: [Hysterectomy] explode all trees
#7	MeSH descriptor: [Morcellation] explode all trees
#8	(hysterectom* OR morcellat* OR sacrocolpopexy):ti,ab,kw
#9	#6 OR #7 OR #8
#10	MeSH descriptor: [Incidental Findings] explode all trees
#11	(occult OR incidental* OR unexpected OR unsuspected OR unanticipated OR undiagnosed OR "inadequately staged" OR "postoperative diagnosis"):ti,ab,kw
#12	#10 OR #11
#13	#5 AND #9 AND #12 (results restricted to Trials [CENTRAL])

Records retrieved:36.

Supplementary Table S3. Study-level risk-of-bias assessment

(A) JBI Critical Appraisal Checklist for Studies Reporting Prevalence Data (studies contributing incidence data, n = 72)

#	Study	Q1	Q2	Q3	Q4	Q5	Q6	Q7	Q8	Q9	Overall
1	Ackenbom 2016 [81]	Y	Y	N	Y	Y	Y	Y	Y	Y	Low
2	Agrawal 2016 [63]	Y	N	N	Y	Y	Y	Y	N	N	Moderate
3	Andiman 2021 [23]	Y	Y	Y	Y	Y	Y	Y	Y	Y	Low
4	Aydın 2016 [82]	Y	Y	N	Y	Y	Y	Y	N	Y	Low
5	Balgobin 2016 [50]	Y	Y	Y	Y	Y	Y	Y	N	Y	Low
6	Barakzai 2023 [85]	Y	Y	N	Y	Y	Y	Y	N	U	Moderate
7	Bharali 2026 [60]	Y	Y	N	Y	Y	Y	Y	N	Y	Low
8	Bojahr 2015 [28]	Y	Y	Y	Y	Y	Y	Y	N	N	Low
9	Bonnar 1970 [74]	Y	U	N	N	Y	Y	U	N	U	High
10	Cao 2024 [19]	Y	Y	Y	Y	Y	Y	Y	Y	Y	Low
11	Chang 2021 [84]	Y	Y	N	Y	Y	Y	Y	Y	Y	Low
12	Channer 1992 [75]	Y	Y	N	N	Y	Y	Y	N	Y	Moderate
13	Damasco 2017 [51]	Y	Y	N	Y	Y	Y	Y	Y	Y	Low
14	Desai 2018 [22]	Y	Y	Y	Y	Y	Y	Y	Y	Y	Low
15	Desai 2019 [25]	Y	Y	Y	Y	Y	Y	Y	Y	Y	Low
16	Ding 2023 [38]	Y	Y	Y	Y	Y	Y	Y	N	Y	Low
17	Ege 2026 [61]	Y	Y	N	Y	Y	Y	Y	N	Y	Low
18	Ehdaivand 2014 [39]	Y	Y	N	Y	Y	Y	Y	N	Y	Low
19	Ekici 2020 [52]	Y	Y	Y	Y	Y	Y	Y	N	Y	Low
20	Frick 2010 [40]	Y	Y	N	Y	Y	Y	Y	Y	Y	Low
21	Gandelsman-Ginis 2025 [88]	Y	Y	N	Y	Y	Y	Y	N	Y	Low
22	Gawron 2018 [17]	Y	Y	N	Y	Y	Y	Y	Y	Y	Low
23	Genc 2015 [49]	Y	U	N	Y	Y	Y	Y	N	U	Moderate
24	Geron 2024 [87]	Y	Y	N	Y	Y	Y	Y	N	Y	Low
25	Gitas 2020 [72]	Y	Y	N	Y	Y	Y	Y	N	Y	Low
26	Graebe 2015 [29]	Y	Y	Y	Y	Y	Y	Y	N	Y	Low
27	Grigoriadis 2015 [80]	Y	Y	N	Y	Y	Y	Y	N	Y	Low
28	Gruner 2025 [36]	Y	Y	Y	Y	Y	Y	Y	N	Y	Low
29	Hagemann 2011 [62]	Y	Y	N	Y	Y	Y	Y	Y	Y	Low

30	Hill 2014 [41]	Y	Y	N	Y	Y	Y	Y	Y	Y	Low
31	Kadıoğlu 2021 [53]	Y	Y	Y	Y	Y	Y	Y	N	Y	Low
32	Kamikabeya 2010 [26] *	Y	Y	Y	U	Y	Y	U	N	U	Moderate
33	Le Neveu 2026 [42] *	Y	Y	Y	U	Y	U	U	U	U	Moderate
34	Li 2026 [90]	Y	Y	Y	Y	Y	Y	Y	N	Y	Low
35	Mahajan 2011 [78]	Y	Y	N	Y	Y	Y	Y	N	Y	Low
36	Mahnert 2015 [30]	Y	Y	Y	Y	Y	Y	Y	Y	Y	Low
37	Marcelle 2026 [37]	Y	Y	N	Y	Y	Y	Y	N	Y	Low
38	Mizrachi 2017 [83]	Y	Y	N	Y	Y	Y	Y	N	Y	Low
39	Mori 2018 [69]	Y	Y	N	Y	Y	Y	Y	N	Y	Low
40	Mühlenbrock 2021 [54]	Y	Y	N	Y	Y	Y	Y	N	Y	Low
41	Ota 2026 [91]	Y	Y	N	Y	Y	Y	Y	N	Y	Low
42	Ouldamer 2014 [43]	Y	Y	Y	Y	Y	Y	Y	Y	Y	Low
43	Parsons 2018 [16]	Y	Y	Y	Y	Y	Y	Y	Y	Y	Low
44	Picerno 2016 [32]	Y	Y	N	Y	Y	Y	Y	Y	Y	Low
45	Raimondo 2024 [73]	Y	Y	N	Y	Y	Y	Y	N	Y	Low
46	Ramm 2012 [79]	Y	Y	N	Y	Y	Y	Y	N	Y	Low
47	Rechberger 2016 [64]	Y	Y	N	Y	Y	Y	Y	N	Y	Low
48	Renganathan 2010 [77]	Y	Y	N	Y	Y	Y	Y	N	Y	Low
49	Rimbach 2017 [67]	Y	Y	N	Y	Y	Y	Y	N	Y	Low
50	Ryu 2023 [86]	Y	Y	N	Y	Y	Y	Y	Y	Y	Low
51	Sabt 2024 [58]	Y	Y	N	Y	Y	Y	Y	N	Y	Low
52	Sanchez Mayorca 2024 [59]	Y	Y	N	Y	Y	Y	Y	N	N	Moderate
53	Serur 2016 [65]	Y	Y	N	Y	Y	Y	Y	N	Y	Low
54	Shim 2018 [70]	Y	Y	N	Y	Y	Y	Y	N	Y	Low
55	Smits 2016 [66]	Y	Y	N	Y	Y	Y	Y	N	Y	Low
56	Sopracordevole 2022 [56]	U	Y	N	Y	Y	Y	Y	N	Y	Moderate
57	Takamizawa 1999 [48]	Y	Y	N	Y	Y	Y	Y	N	Y	Low
58	Tan 2015 [31]	Y	Y	N	Y	Y	Y	Y	N	Y	Low
59	Tchartchian 2019 [71]	Y	Y	N	Y	Y	Y	Y	N	Y	Low
60	Theben 2013 [27]	Y	Y	Y	Y	U	Y	Y	N	N	Moderate
61	Topdagi 2020 [44]	Y	Y	N	Y	Y	Y	Y	N	Y	Low
62	Vallabh-Patel 2016 [45]	Y	Y	N	Y	Y	Y	Y	N	Y	Low
63	Von Bargen 2017 [68]	Y	Y	N	Y	Y	Y	Y	N	Y	Low

64	Wagner 2019 [12]	Y	Y	Y	Y	Y	Y	Y	Y	Y	Low
65	Wan 2013 [46]	Y	Y	N	Y	Y	Y	Y	N	Y	Low
66	Wankhade 2023 [57]	Y	U	N	N	Y	Y	U	N	U	High
67	Wasson 2017 [33]	Y	Y	N	Y	Y	Y	Y	N	Y	Low
68	Weigel 2025 [89]	Y	Y	N	Y	Y	Y	Y	N	Y	Low
69	Yadav 2021 [55]	Y	N	N	Y	Y	Y	Y	N	U	Moderate
70	Yildiz 2021 [47]	Y	Y	Y	Y	Y	Y	Y	N	Y	Low
71	Yin 2004 [76]	Y	Y	N	Y	Y	Y	Y	N	Y	Low
72	Yuk 2021 [24]	Y	Y	Y	Y	Y	U	U	N	Y	Moderate

Q1 sample frame appropriate; Q2 participants sampled appropriately; Q3 adequate sample size (≥1277 hysterectomies; ±0.3 percentage points at expected incidence 0.3%); Q4 subjects and setting described; Q5 sufficient coverage of identified sample; Q6 valid methods for identification of the condition; Q7 condition measured in a standard, reliable way; Q8 appropriate statistical analysis (incidence with 95% CI); Q9 adequate response rate/missing data. Y yes; N no; U unclear. Overall: low ≥7, moderate 4–6, high ≤3 items met. *Full text not retrieved; appraised from the published abstract. Inter-rater agreement: 647/648 item ratings (99.8%; κ = 0.996).

(B) QUIPS (studies contributing prognostic or management data, n = 8)

Study	D1	D2	D3	D4	D5	D6	Overall	Contribution
Bojahr 2015 [28]	Low	Moderate	Low	Low	Moderate	Low	Moderate	Follow-up after morcellated occult malignancy
Wagner 2019 [12]	Low	Low	Low	Low	Moderate	Low	Moderate	Stage and outcome of occult EC
Xu 2019 [9]	Low	Low	Low	Low	Low	Low	Low	Morcellation vs abdominal, mortality
Yuk 2021 [24]	Low	Low	Moderate	Low	Moderate	Low	Moderate	Laparoscopy vs laparotomy, survival
Gruner 2025 [36]	Moderate	Low	Low	Low	Moderate	Low	Moderate	Occult vs preoperatively diagnosed EC, survival
Thomas 2018 [34]	Moderate	Moderate	Moderate	Low	High	Moderate	Moderate	Completion staging, upstaging
Barquet-Muñoz 2025 [35]	Moderate	Moderate	Low	Low	Moderate	Moderate	Moderate	Complementary lymphadenectomy, survival

Einstein 2008 [92]	Moderate	Moderate	Moderate	Low	High	Moderate	Moderate	Management after incidental malignancy

D1 study participation; D2 study attrition; D3 prognostic factor/exposure measurement; D4 outcome measurement; D5 study confounding and adjustment; D6 statistical analysis and reporting. EC, endometrial carcinoma.

Supplementary Table S4. Sensitivity of pooled incidence estimates to the random-effects model

Method	Overall<br>% (95% CI)	All-benign<br>% (95% CI)	Morcellation<br>% (95% CI)	POP<br>% (95% CI)
Occult endometrial carcinoma (k = overall 68, all-benign 25, morcellation 19, POP 24)				
**Freeman–Tukey, DerSimonian–Laird (primary analysis)**	**0.33 (0.26–0.40)**	**0.33 (0.24–0.43)**	**0.22 (0.13–0.33)**	**0.43 (0.32–0.55)**
Freeman–Tukey, restricted maximum likelihood	0.33 (0.27–0.40)	0.32 (0.23–0.43)	0.22 (0.13–0.33)	0.43 (0.33–0.54)
Freeman–Tukey, Paule–Mandel	0.33 (0.27–0.40)	0.32 (0.23–0.43)	0.21 (0.13–0.31)	0.43 (0.32–0.56)
Freeman–Tukey, DerSimonian–Laird + HKSJ	0.33 (0.27–0.40)	0.33 (0.23–0.44)	0.22 (0.13–0.33)	0.43 (0.32–0.56)
Logit, DerSimonian–Laird (0.5 continuity correction)	0.37 (0.31–0.44)	0.37 (0.29–0.47)	0.26 (0.17–0.39)	0.49 (0.40–0.61)
Binomial–normal GLMM	0.28 (0.22–0.35)	0.29 (0.20–0.39)	0.17 (0.09–0.28)	0.41 (0.30–0.52)
GLMM between-study SD (τ, logit scale)	0.56	0.57	0.57	0.16
All occult uterine malignancy (endometrial carcinoma + sarcoma) (k = overall 66, all-benign 23, morcellation 19, POP 24)				
**Freeman–Tukey, DerSimonian–Laird (primary analysis)**	**0.54 (0.45–0.64)**	**0.67 (0.54–0.82)**	**0.43 (0.27–0.62)**	**0.46 (0.36–0.58)**
Freeman–Tukey, restricted maximum likelihood	0.54 (0.45–0.64)	0.67 (0.53–0.83)	0.42 (0.28–0.59)	0.47 (0.38–0.57)
Freeman–Tukey, Paule–Mandel	0.54 (0.46–0.63)	0.67 (0.54–0.82)	0.41 (0.28–0.57)	0.46 (0.34–0.59)
Freeman–Tukey, DerSimonian–Laird + HKSJ	0.54 (0.46–0.63)	0.67 (0.54–0.83)	0.43 (0.28–0.61)	0.46 (0.35–0.59)
Logit, DerSimonian–Laird (0.5 continuity correction)	0.56 (0.48–0.65)	0.65 (0.54–0.80)	0.44 (0.30–0.67)	0.52 (0.44–0.61)
Binomial–normal GLMM	0.48 (0.39–0.57)	0.61 (0.47–0.77)	0.35 (0.22–0.52)	0.47 (0.34–0.55)
GLMM between-study SD (τ, logit scale)	0.47	0.43	0.53	0.03

Values are pooled incidence, % (95% CI). Primary analysis shown in bold. DL, DerSimonian–Laird; HKSJ, Hartung–Knapp–Sidik–Jonkman adjustment (t-distribution with k−1 degrees of freedom); GLMM, generalized linear mixed model (random-intercept logistic regression on the exact binomial likelihood, fitted by maximum likelihood with adaptive Gauss–Hermite quadrature; 95% CIs by profile likelihood; no continuity correction). The GLMM estimate represents the median study-level incidence on the logit scale and is therefore slightly lower than marginal estimates from transformed-proportion models. Formal tests for subgroup differences (all-benign vs morcellation-specific vs POP), occult endometrial carcinoma: test for subgroup differences (Freeman–Tukey random effects) $p = 0.03$; GLMM likelihood-ratio test ($\chi^2 = 6.75$, df = 2) $p = 0.03$; random-effects meta-regression with common between-study variance $p = 0.08$. All occult uterine malignancy: GLMM likelihood-ratio test ($\chi^2 = 7.53$, df = 2) $p = 0.02$.
